# Efficacy of Panitumumab and Cetuximab in Patients with Colorectal Cancer Previously Treated with Bevacizumab; a Combined Analysis of Individual Patient Data from ASPECCT and WJOG6510G

**DOI:** 10.3390/cancers12071715

**Published:** 2020-06-28

**Authors:** Hiroya Taniguchi, Takeharu Yamanaka, Daisuke Sakai, Kei Muro, Kentaro Yamazaki, Susumu Nakata, Hiroyuki Kimura, Paul Ruff, Tae Won Kim, Marc Peeters, Timothy Price

**Affiliations:** 1 Department of Gastroenterology and Gastrointestinal Oncology, National Cancer Centre Hospital East, Kashiwa 277-8577, Japan; 2 Department of Clinical Oncology, Aichi Cancer Centre, Nagoya 464-8681, Japan; kmuro@aichi-cc.jp; 3 Department of Biostatistics, Yokohama City University School of Medicine, Yokohama 236-0004, Japan; yamanaka@yokohama-cu.ac.jp; 4 Frontier Science for Cancer and Chemotherapy, Osaka University Graduate School of Medicine, Suita 565-0871, Japan; dsakai@cfs.med.osaka-u.ac.jp; 5 Division of Gastrointestinal Oncology, Shizuoka Cancer Centre, Shizuoka 411-8777, Japan; k.yamazaki@scchr.jp; 6 Department of Clinical Oncology, Kyoto Pharmaceutical University, Kyoto 607-8414, Japan; snakata@mb.kyoto-phu.ac.jp; 7 Department of Analytical and Bioinorganic Chemistry, Kyoto Pharmaceutical University, Kyoto 607-8414, Japan; hkimura@mb.kyoto-phu.ac.jp; 8 University of Witwatersrand MRC Common Epithelial Cancers Research Centre, Johannesburg 2193, South Africa; pruff@iafrica.com; 9 Department of Oncology, Asan Medical Centre, University of Ulsan College of Medicine, Seoul 05505, Korea; twkimmd@amc.seoul.kr; 10 Department of Oncology, U.Z.A. University Hospital Antwerp, 2650 Edegem, Belgium; marc.peeters@uza.be; 11 Department of Medical Oncology, Queen Elizabeth Hospital and University of Adelaide, 5011 Woodville, Australia; Timothy.Price@sa.gov.au

**Keywords:** colorectal cancer, anti-EGFR therapy, panitumumab, cetuximab

## Abstract

*Background:* Phase-III ASPECCT and randomised phase-II WJOG6510G trials demonstrated the noninferiority of panitumumab, when compared with cetuximab, for overall survival in patients with chemotherapy-refractory wild-type *KRAS* exon 2 metastatic colorectal cancer. *Methods:* The subgroup that received bevacizumab either prior to panitumumab or cetuximab monotherapy (ASPECCT) or in combination with irinotecan (WJOG6510G) was included. Multivariate Cox models were created, including the treatment arms as covariates together with patient, disease and treatment characteristics. *Results:* We included 185 and 189 patients in the panitumumab and cetuximab arms, respectively. The median overall survival was 12.8 and 10.1 months [*p* = 0.0031; log-rank test, stratified by trial; hazard ratio (HR), 0.72; 95% confidence interval (CI), 0.58–0.90], and the median progression-free survival was 4.7 and 4.1 months, in the panitumumab and cetuximab arms, respectively (*p* = 0.0207; HR, 0.79; 95% CI, 0.64–0.97). The treatment regimen was an independent prognostic factor of overall survival (adjusted HR, 0.69; 95% CI, 0.54–0.87; *p* = 0.0013). *Conclusions:* Panitumumab significantly prolonged the overall survival and progression-free survival, when compared with cetuximab in the cohort that previously received bevacizumab in the included studies. *Clinical Trial Registration:* ASPECCT trial registered with ClinicalTrials.gov (NCT01001377) and WJOG6510G trial registered with UMIN-CTR (UMIN000006643).

## 1. Introduction

Colorectal carcinoma (CRC) is the third leading type of cancer, and cause of cancer deaths, worldwide. Metastatic CRC (mCRC) develops in approximately half of all patients diagnosed with CRC, and the resulting poor prognosis is a potent driver of ongoing efforts to find treatments to improve patient outcomes. Panitumumab, a fully human monoclonal antibody that targets the epidermal growth factor receptor (EGFR), and cetuximab, a chimeric anti-EGFR antibody, have demonstrated clinical efficacy in patients with chemotherapy-refractory wild-type *KRAS* exon 2 mCRC. In the phase-III CO.17 study, cetuximab monotherapy improved overall survival (OS) and progression-free survival (PFS), versus best supportive care (BSC), in patients with wild-type *KRAS* exon 2 tumours [1,2]. In a cohort of these patients with mCRC, the phase-III 20020408 study showed that BSC plus panitumumab improved PFS compared with BSC alone. However, no statistically significant benefit was seen with panitumumab monotherapy for OS in the 20020408 study. This was probably because of patient crossover from BSC alone to BSC plus panitumumab after disease progression. Indeed, the subsequent 20100007 phase-III study that lacked this crossover showed a clear improvement of OS with panitumumab plus BSC, versus BSC alone, in these patients with chemotherapy-refractory wild-type *KRAS* exon 2 mCRC.

The panitumumab or cetuximab monotherapy (ASPECCT) was the first head-to-head, randomised, phase-III study of panitumumab versus cetuximab for the treatment of chemotherapy-refractory wild-type *KRAS* exon 2 metastatic colorectal cancer (February 2010 to July 2012). The primary analysis demonstrated that panitumumab was non-inferior to cetuximab, and that both agents provided a similar overall survival benefit in this population [3]. Interestingly, 25% of the patients had previously received treatment with bevacizumab, and the median overall survival appeared better in this subgroup among patients treated with panitumumab (11.3 months) than the overall survival rate for those treated with cetuximab [9.8 months; hazard ratio (HR), 0.75; 95% confidence interval (CI), 0.58–0.97]. After adjustment for baseline covariates, the HR for the overall survival between panitumumab and cetuximab was 0.65 (95% CI, 0.49–0.85) [4].

The WJOG6510G trial was the second head-to-head, randomised, phase-II study of panitumumab versus cetuximab (December 2011 to September 2014). In this trial, each agent was given in combination with irinotecan. This was based on the randomised phase-II BOND study demonstrating that cetuximab produced a higher response rate when given in combination with irinotecan [5]. Notably, the median progression-free survival was 5.4 months in the panitumumab arm, and 4.3 months in the cetuximab arm (HR, 0.68; 95% CI, 0.47–0.99; *p* < 0.001 for noninferiority, *p* = 0.040 for superiority); the corresponding median overall survival durations were 14.9 and 11.5 months, respectively (HR, 0.68; 95% CI, 0.46–1.02; *p* = 0.06) [6]. Most patients (97%) had received chemotherapy that included bevacizumab.

We speculated that panitumumab may be better than cetuximab for patients with prior bevacizumab treatment, based on the results from the ASPECCT and WJOG6510G head-to-head studies. However, although ASPECCT had a large sample size overall (*n* = 1010), few patients had previously received bevacizumab (25%); by contrast, the WJOG6510G trial had a small overall sample size (*n* = 121), but a large group had previously received bevacizumab (97%). Therefore, we aimed to collect data for individual patients enrolled in each trial, to clarify whether panitumumab has better efficacy than cetuximab among patients who have previously received bevacizumab.

## 2. Results

### 2.1. Participants

**Figure 1:**
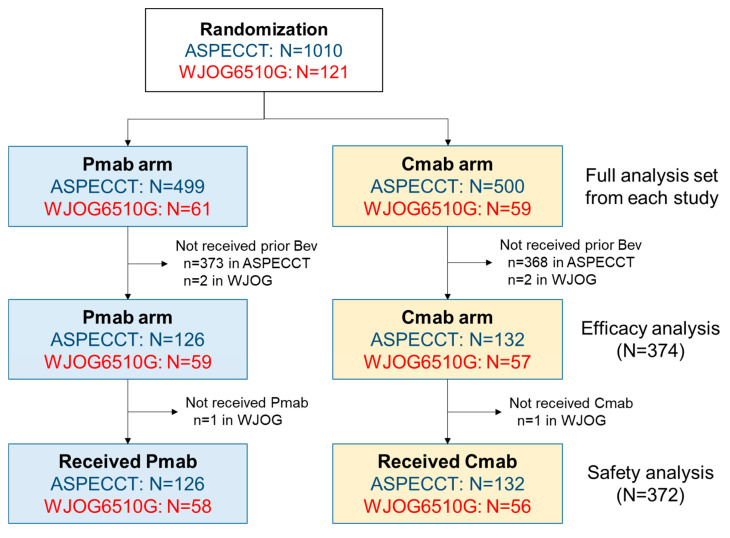
Study population. The panitumumab or cetuximab monotherapy (ASPECCT)was a phase-III trial of panitumumab versus cetuximab monotherapy. The WJOG6510G was a randomised phase-II trial of panitumumab plus irinotecan, versus cetuximab plus irinotecan. Abbreviations: Bev, bevacizumab; Cmab, cetuximab; Pmab, panitumumab.

Figure 1 summarises the case enrolment for pooled analysis of the two trials. Ultimately, 374 patients were included in the efficacy analysis (panitumumab, *n* = 185; cetuximab, *n* = 189), and two were excluded from the safety analysis (one per arm) because they did not receive the study treatment. The baseline characteristics of patients in the panitumumab and cetuximab treatment arms are summarised in Table 1. Compared with the cetuximab arm, the panitumumab arm had higher numbers of males (68.6% vs. 59.3%), patients with at least two metastatic sites (38.9% vs. 31.2%), and patients with higher median carcinoembryonic antigen levels (90.50 vs. 55.70 ng/mL). However, there were no differences between arms in age, Eastern Cooperative Oncology Group (ECOG) performance status, tumour location (colon/rectum), prior surgery (yes/no), liver-limited disease or post-progression anti-tumour therapy.

### 2.2. Pooled Overall Survival and Progression-Free Survival Analyses

**Figure 2:**
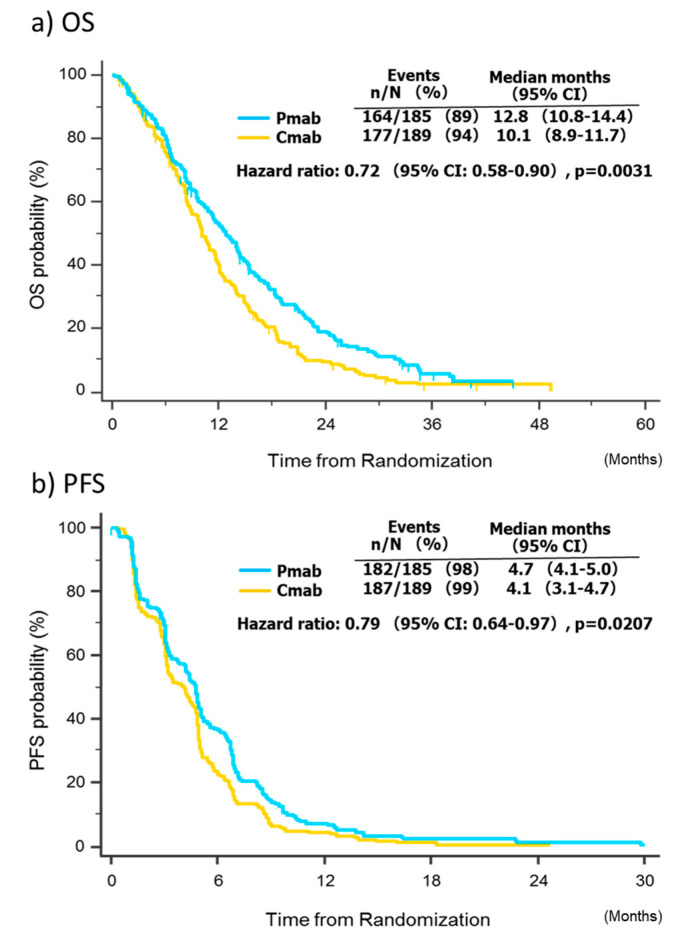
Survival analysis. Abbreviations: *CI*, confidence interval; Cmab, cetuximab; Pmab, panitumumab; OS, overall survival; PFS, progression-free survival.

The pooled overall survival analysis was based on 341 events among the 374 patients (91.2%). The median overall survival was 12.8 months (95% CI, 10.8–14.4) in the panitumumab group, compared with 10.1 months (95% CI, 8.9–11.7) in the cetuximab group, with an adjusted HR of 0.72 (95% CI 0.58–0.90; *p* = 0.0031; Figure 2a). The pooled progression-free survival analysis was based on 369 events among 374 patients (98.7%). The median progression-free survival was 4.7 months (95% CI, 4.1–5.0) in the panitumumab group, compared with 4.1 months (95% CI, 3.1–4.7) in the cetuximab group, and the adjusted HR was 0.79 (95% CI, 0.64–0.97; *p* = 0.021; Figure 2b).

### 2.3. Pooled Univariate and Multivariate Analyses of Factors Affecting Survival

Four factors were associated with overall survival in the univariate analysis: ECOG performance status, number of metastatic sites, carcinoembryonic antigen level and treatment group (Table 2). All four factors remained significantly associated with survival in the multivariate analysis. Of note, the risk of death was significantly decreased among patients in the panitumumab group, compared with those in the cetuximab group, with an HR of 0.69 (95% CI, 0.54–0.87; *p* = 0.0013).

### 2.4. Pooled Analysis of Treatment Response

In patients with measurable disease at baseline, there was no difference in the proportion of patients who achieved an objective response between the panitumumab arm (42 patients; 22.7%; 95% CI, 16.7–28.7) and the cetuximab arm (30 patients; 15.9%; 95% CI, 10.6–21.1) (*p* = 0.11). There was also no difference in the disease control rate between the panitumumab (72.4%) and cetuximab (67.2%) treatment arms (*p* = 0.31).

### 2.5. Pooled Analysis of Treatment Safety

The safety analysis set included 184 patients in the panitumumab arm and 188 in the cetuximab arm. Table 3 summarises the experience of adverse events related to anti-EGFR therapy in each arm. Although the incidence of skin toxicity was not different between the panitumumab arm (89.7% of any grade, 13.6% of grade ≥ 3) and the cetuximab arm (87.8% of any grade, 9.6% of grade ≥ 3), infusion reactions were more common with cetuximab (8.5% of any grade) than with panitumumab (1.1% of any grade), and hypomagnesaemia was more common with panitumumab (47.0% of any grade) than with cetuximab (32.0% of any grade).

## 3. Discussion

Form our post-hoc combined analysis, we infer an improved survival outcome with panitumumab compared to with cetuximab in patients with metastatic colorectal cancer who were previously treated with bevacizumab, by pooling individual patient data from two head-to-head trials. The benefits of panitumumab for overall survival were also confirmed by a multivariate analysis that included other key prognostic factors, which provided a cleaner and less ambiguous analysis. Generally, the reliability of evidence from a subgroup analysis is inadequate in a single, randomised controlled trial, because of multiplicity and a lack of power. Performing a combined analysis based on individual patient data from the ASPECCT and WJOG6510G trials remedied these issues. Although there was some variation in the statistical considerations and design (irinotecan use), this approach was feasible and valid because the trial designs and inclusion criteria were almost identical.

Some clinical data indicate that the efficacy of cetuximab may be reduced for patients with wild-type KRAS exon 2 metastatic colorectal cancer who previously received bevacizumab. For example, an Italian randomised study compared the use of second-line irinotecan plus cetuximab, followed by third-line fluorouracil, leucovorin, and oxaliplatin (FOLFOX) (or a reverse sequence) after first-line chemotherapy, with the use of FOLFIRI plus bevacizumab, for patients with metastatic colorectal cancer. This demonstrated that overall survival was better in the latter arm with the reverse sequence (median overall survival: 12.3 vs. 18.6 months) [7]. In the PRODIGE18 trial, which compared bevacizumab or cetuximab plus chemotherapy after progression with bevacizumab plus chemotherapy, a nonsignificant difference was shown that favoured continuing bevacizumab and chemotherapy (median overall survival, 15.8 vs. 10.4 months; HR, 0.69; 95% CI, 0.46–1.04; *p* = 0.08) [8]. Other research has indicated that the efficacy of panitumumab may not be particularly affected by prior bevacizumab treatment. For example, in the randomised phase-II WJOG6210G study, treatment with FOLFIRI plus panitumumab showed favourable survival when compared with FOLFIRI plus bevacizumab, as a second-line chemotherapy for disease refractory to first-line chemotherapy containing oxaliplatin and bevacizumab (median overall survival, 16.2 vs. 13.4 months; HR, 1.16; 95% CI, 0.76–1.77) [9]. Although we should interpret this cautiously, because of the indirect comparison, panitumumab may confer survival benefits over cetuximab for patients with metastatic colorectal cancer who have received bevacizumab. This is consistent with the findings of the pooled analysis of the ASPECCT and WJOG6510G trials.

A potential mechanism for the different efficacies of panitumumab and cetuximab may be their different affinities for binding to the EGFR. Panitumumab has been reported to have a 1- to 2-log higher affinity than cetuximab, but a binding epitope that is similar [10]. Some basic research has shown that cetuximab adequately blocks low-affinity ligands (e.g., AREG and EREG) from binding to EGFR, but that it cannot block activation of the EGFR pathway by high-affinity ligands (e.g., EGF, BTC and TGFα) [11]. In contrast to this, panitumumab effectively inhibits both low- and high-affinity ligand-driven ERK signaling [12]. The higher incidence of hypomagnesaemia associated with panitumumab use may also be explained by its different affinity to EGFR, because anti-EGFR antibodies are accompanied by renal magnesium wasting, due to the blockage of the EGF–EGFR pathway in the basolateral tubular epithelium [13]. Colorectal cancer growth depends on low-affinity ligands [14], yet panitumumab and cetuximab have shown similar efficacy via indirect comparisons in a first-line setting [15,16], including the subgroup who did not previously receive bevacizumab in the ASPECCT trial. However, bevacizumab reduces vascular density and causes hypoxia in the tumour [17], which may induce angiogenic factors, such as HB-EGF, BTC and EGF, which are also high-affinity EGFR ligands [18]. Hypoxic tumour regions had lower distribution in a mouse model treated with cetuximab [19]. Because panitumumab has a higher affinity to EGFR, it may be able to bind EGFR under hypoxic conditions after bevacizumab therapy. We believe that this hypothesis explains the difference in outcomes between panitumumab and cetuximab among patients who have received bevacizumab, but more basic research is needed into the underlying biological mechanisms.

There were some limitations to our study, mostly related to the use of retrospective data from two prospective, randomised controlled trials. First, wild-type *KRAS* status was defined as a lack of mutation seen in exon 2, and we did not consider the effects of other significantly rare *RAS* and *BRAF* mutations. However, in the additional biomarker analysis of the WJOG6510G trial, patients with extended *RAS* mutation or *BRAF* V600E mutations were well balanced between both arms, whereas the survival advantage of panitumumab was also shown in the wild-type *RAS* subpopulation. Secondly, primary tumour localisation (right/left), a key predictive factor of anti-EGFR therapy [20], was also excluded from the pooled analysis because there was a lack of information in the ASPECCT cohort. That said, most patients randomised in the WJOG6510G trial (87%) had left-sided tumours, meaning that this also applied to most patients in the pooled data. Recently, it was shown that adding panitumumab or cetuximab to oxaliplatin- or irinotecan-based chemotherapy produced a clear survival benefit when used as a first-line therapy, with this approach recommended for patents with left-sided metastatic colorectal cancer and wild-type *RAS*. Therefore, the majority of patients with left-sided colorectal cancer will be treated with anti-EGFR therapy as the first-line therapy. Our trial situation with patients receiving anti-EGFR therapy as the later-line treatment may not represent the recent typical clinical scenario. Soon, a re-challenge strategy using anti-EGFR therapy may be active in patients with *RAS* and *BRAF* wild-type tumours, who have acquired resistance to first-line cetuximab-based therapies [21]. Though the clinical features and tumour biology of patients who have become resistant to bevacizumab in the second-line may be different to those of first-line bevacizumab refractory patients, the survival benefits of panitumumab may be superior to cetuximab in such a re-challenge situation in the left-sided tumour. However, this is only speculation, and further investigation is required.

In conclusion, our combined analysis, using individual patient data from the ASPECCT and WJOG6510G trials, confirms that panitumumab has a survival advantage over cetuximab in patients with metastatic colorectal cancer, who have previously received bevacizumab. The unexpected findings should lead to further exploration of the underlying mechanisms, and may be relevant to practice guidance when considering anti-EGFR therapy as a third-line option. Studies are also needed to confirm the current outcomes.

## 4. Materials and Methods 

### 4.1. Study Design

Detailed information has previously been reported concerning the patient inclusion criteria, study design and treatment schedules of the ASPECCT trial. The WJOG6510G trial used similar inclusion criteria. Briefly, patients were eligible if they met the following criteria: histologically confirmed unresectable metastatic colorectal cancer; refractory or intolerant to fluorouracil-, oxaliplatin- and irinotecan-based chemotherapy; wild-type *KRAS* exon 2 based on local assessment ECOG performance status, 0–2; presence of measurable disease, as defined by the Response Evaluation Criteria In Solid Tumors version 1.1 (RECIST v1.1); and adequate hematologic, renal, hepatic and metabolic function. Patients who had previously been treated with anti-EGFR antibodies were excluded. Both trials were conducted according to the ethical principles of the Declaration of Helsinki, the study protocols were approved by an appropriate institutional review board, and all patients provided written informed consent. The ASPECCT trial was registered with ClinicalTrials.gov (NCT01001377) and the WJOG6510G trial was registered with UMIN-CTR (UMIN000006643).

### 4.2. Treatment

Patients were randomised on a 1:1 basis to receive either panitumumab or cetuximab intravenously. Panitumumab (6 mg/kg) was given on day 1 of each 14-day cycle, whereas cetuximab was given as an initial dose of 400 mg/m^2^ followed by 250 mg/m^2^ on day 1 of each 7-day cycle. In the WJOG6510G trial, 150 mg/m^2^ irinotecan was also given intravenously every 2 weeks. The starting dose of irinotecan could be reduced to 120 or 100 mg/m^2^ if patients had required a dose reduction of irinotecan during previous treatment. In both trials, patients in the cetuximab arm received premedication (an H1 antagonist with or without dexamethasone) before infusion. No premedication was required with panitumumab. Treatment continued until disease progression, intolerability or withdrawal of consent occurred.

### 4.3. Efficacy and Safety Assessments

In the ASPECCT trial, computed tomography or magnetic resonance imaging of the abdomen, pelvis and chest was assessed after 6 weeks of treatment and approximately every 8 weeks thereafter. In the WJOG6510G trial, tumour assessments were repeated every 8 weeks from randomisation up to discontinuation of the protocol treatment. Responses were assessed by each investigator based on Response Evaluation Criteria in Solid Tumours version 1.1, and there was no central review of response in either trial. Laboratory tests were performed at screening and baseline, and they were repeated at least once every 4 weeks in the ASPECCT trial, or every 2 weeks in the WJOG6510G trial. Adverse events were graded according to the National Cancer Institute Common Terminology Criteria for Adverse Events v3.0 in the ASPECCT trail (skin-related or nail-related toxicities were graded with some modification), whereas the CTCAE v4.0 was used to evaluate adverse events in the WJOG6510G trial.

### 4.4. Outcomes and Definitions

In the pooled analysis, we evaluated the overall survival and the progression-free survival as the main outcome variables. The overall survival was defined as the time from randomisation to death from any cause, whereas the progression-free survival was defined as the time from randomisation to evidence of disease progression, according to Response Evaluation Criteria in Solid Tumours version 1.1, or death (whichever occurred first). The response rate was the number of patients who achieved a best overall response of complete or partial response. The disease control rate was the number of patients who achieved a best overall response of complete response, partial response or stable disease. For the safety analyses, we only assessed anti-EGFR-related toxicities: skin toxicities (e.g., rash, dermatitis acneiform, dry skin and paronychia), electrolyte abnormalities (hypomagnesaemia, hypokalaemia and hypocalcaemia), infusion reactions, intestinal lung disorders and stomatitis or mucosal inflammation.

The primary efficacy analysis followed a modified intention-to-treat principle. The primary analysis set included all randomised patients in each study who were eligible, received at least one dose of panitumumab or cetuximab, and received bevacizumab prior to enrolment. The safety analysis set included all randomised patients in each study who received bevacizumab prior to enrolment and at least one dose of panitumumab or cetuximab.

### 4.5. Statistical Analysis of the Pooled Data

In the pooled analysis, we used the final data set for each trial: September 15, 2014, for the ASPECCT trial and March 31, 2017, for the WJOG6510G trial. The progression-free survival was summarised as Kaplan–Meier estimates by treatment arm after combing data for the two studies. The estimated median survival times and survival proportions at 6 and 12 months were calculated with their 95% CIs. A Cox regression model, stratified by study, was used to compare progression-free survival between the two arms by HR and two sided 95% CIs. Adjusted HRs were estimated on the basis of a multivariate stratified Cox regression model, adjusting for important prognostic factors whose distributions were imbalanced between the two arms. Besides the treatment arms, we included any baseline patient, disease or treatment characteristics associated with overall survival, at the *p* ≤ 0.1 significance level by univariate analysis, as covariates in the multivariate Cox models.

Baseline demographic and clinical variables were compared by chi-square tests for heterogeneity, when categorical, and by Wilcoxon Mann–Whitney U-test when continuous. Missing values were handled by a single imputation technique. Estimates of PFS and OS were calculated according to the Kaplan–Meier product-limit method. In the univariate and multivariate analyses, odds ratios and HRs were calculated by logistic regression or Cox proportional hazard modeling, respectively. All statistical tests were two sided, with p-values of ≤ 0.05 considered statistically significant. No adjustment was made for multiple comparisons. Statistical analyses were performed with SAS version 9.2 (SAS Institute, Inc., Cary, NC, USA) by the ASPECCT-WJOG6510G Pooled Analysis Project Team, using individual patient data shared by Amgen and the West Japan Oncology Group.

## 5. Conclusions

Panitumumab significantly prolonged the overall survival and progression-free survival, compared with cetuximab, in the cohort that previously received bevacizumab in the included studies.

## Figures and Tables

**Table 1:** Patient characteristics.

Characteristics, *n* (%).		Panitumumab		Cetuximab		
		*N* = 185		*N* = 189		*p*-Value
Age	Median (Range)	61	(32–80)	62	(26–82)	0.84
	<65 years	119	(64.3%)	117	(61.9%)	0.63
	≥65 years	66	(35.7%)	72	(38.1%)	
Sex	Male	127	(68.6%)	112	(59.3%)	0.059
	Female	58	(31.4%)	77	(40.7%)	
ECOG PS	0	81	(43.9%)	83	(43.8%)	
	1	92	(48.7%)	92	(49.7%)	0.93
	2	12	(6.5%)	14	(7.4%)	
Tumour location	Colon	114	(61.6%)	120	(63.5%)	0.71
	Rectum	71	(38.4%)	69	(36.5%)	
Prior surgery	Yes	168	(90.8%)	172	(91.0%)	1.0
	No	17	(9.2%)	17	(9.0%)	
Number of metastatic sites	1–2	113	(61.1%)	130	(68.8%)	0.13
	≥3	72	(38.9%)	59	(31.2%)	
Liver metastasis only	Yes	24	(13.0%)	30	(15.9%)	0.46
	No	161	(87.0%)	159	(84.1%)	
CEA	Median (ng/mL)	90.50		55.70		0.028
	<50	71	(39.9%)	90	(48.9%)	0.091
	≥50	107	(60.1%)	94	(51.1%)	

Abbreviations: ECOG PS, Eastern Cooperative Oncology Group performance status; CEA, Carcinoembryonic antigen.

**Table 2:** Results of the univariate and multivariate analyses for OS.

**Univariate analysis for OS**				
**Factor**	**Category**	***N***	**Median OS (m)**	***p*** **-Value**
Age	<65/≥65	236/138	10.09/13.44	0.077
Sex	F/M	135/239	10.87/11.63	0.72
ECOG PS	0–1/2	348/26	11.92/4.62	<0.0001
Tumour location	Colon/Rectum	234/140	11.27/11.93	0.42
Prior surgery	No/Yes	34/340	8.71/11.63	0.29
No. of mets	1–2/≥3	243/131	12.81/8.71	0.00014
Liver met only	No/Yes	320/54	11.01/12.35	0.67
CEA	<50/≥50 (ng/mL)	161/201	13.27/10.51	0.0036
Study	ASPECCT/WJOG	258/116	10.35/12.81	0.29
Regimen	Cmab/Pmab	189/185	10.09 / 13.27	0.0077
**Multivariate analysis for OS**				
**Factor**	**Category**	**HR**	**95%CI**	***p*-Value**
ECOG PS	0–1 vs. 2	2.50	1.62–3.84	<0.0001
No. of mets	1–2 vs. ≥3	1.57	1.22–2.00	0.00030
CEA	<50 vs. ≥50 (ng/mL)	1.32	1.03–1.67	0.023
Regimen	Cmab vs. Pmab	0.69	0.54–0.87	0.0013

Abbreviations: ECOG PS, Eastern Cooperative Oncology Group performance status; No., number; mets; metastatic sites; CEA; Cmab, cetuximab; Pmab, panitumumab.

**Table 3:** Anti-EGFR-related adverse events.

	CTCAE v 4.0	Panitumumab		Cetuximab		*p*-Value ^a^
		*N* = 184		*N* = 188		
Skin toxicity ^b^	Any grade	165	(89.7%)	165	(87.8%)	0.625
	Grade ≥ 3	25	(13.6%)	18	(9.6%)	0.258
Infusion reaction ^c^	Any grade	2	(1.1%)	16	(8.5%)	0.0054
	Grade ≥ 3	0	(0%)	4	(2.1%)	0.2440
Hypomagnesemia	Any grade	86	(47.0%) ^d^	60	(32.0%)	0.0040
	Grade ≥ 3	22	(12.0%) ^d^	7	(3.7%)	0.0033

National Cancer Institute Common Terminology Criteria for Adverse Events (NCI CTCAE) v3.0 was used. ^a^ Fisher’s exact test; ^b^ Rash, acne, skin toxicity, dermatitis, dermatitis acneiform, erythema; ^c^ Infusion reaction, hypersensitivity, anaphylactic reaction, cytokine release syndrome; ^d^ One patient was excluded due to missing data of serum magnesium levels. EGFR: epidermal growth factor receptor.
